# How to nudge students toward healthier snacks? Consumer neuroscience insights on multisensory nudge interventions in university vending machines

**DOI:** 10.1371/journal.pone.0325804

**Published:** 2025-06-26

**Authors:** Chiara Casiraghi, Simone Chiarelli, Giuseppina Gifuni, Alessandro Fici, Marco Bilucaglia, Alessandra Cecilia Jacomuzzi, Valeria Micheletto, Margherita Zito, Vincenzo Russo

**Affiliations:** 1 Behavior and Brain Lab IULM – Neuromarketing Research Center, Università IULM, Milan, Italy; 2 Department of Business, Law, Economics and Consumer Behaviour “Carlo A. Ricciardi”, Università IULM, Milan, Italy; 3 Department of Philosophy and Cultural Heritage, Ca’ Foscari University of Venice, Venice, Italy; Lusofona University of Humanities and Technologies: Universidade Lusofona de Humanidades e Tecnologias, PORTUGAL

## Abstract

Nudge has proven effective in promoting healthier eating, especially in academic environments. However, its application in vending machines has not been extensively studied yet, with existing studies focusing on choice and overlooking the emotional and cognitive responses to these interventions. Our research explored how visual and olfactory nudges (and a combination of both) can encourage healthier choices in university vending machines, and examined the related emotional and cognitive reactions, adopting a consumer neuroscience approach. It encompassed three distinct analysis levels: behavioral (snack choice), neurophysiological (emotional and cognitive reactions assessed through skin conductance and electroencephalography), and attentional (visual attention on snacks evaluated via eye-tracking). The findings revealed that while visual and olfactory nudges, when considered individually, were associated with an increased likelihood of making healthier choices, their combined effect was not significant. Skin conductance indicated that olfactory and combined nudge interventions induced relaxation, potentially promoting healthier choices. Electroencephalography results suggested a reduced motivational approach toward snacks when both nudges were combined. Eye-tracking indicated that individuals pay heightened attention to healthy snacks when positioned at eye level, however, this can also be affected by the nudge condition. Our findings suggest that implementing visual or olfactory nudges alone may promote healthier choices in vending machines, whereas combining them may lessen their impact, and corroborate the importance of positioning target products in locations where they are readily visible to consumers. These insights enhance our understanding of the emotional, cognitive, and attentional components behind nudge interventions and offer practical strategies for promoting healthier eating habits.

## Introduction

The increasing prevalence of obesity among university students has become a significant public health issue [1,2], highlighting the importance of educational environments in implementing targeted interventions against overweight and obesity [3]. Several studies have indeed shown that students tend to gain weight during their initial university years [4–6] when they are more likely to make poor dietary choices, particularly in relation to the consumption of food from vending machines [7,8].

In light of these considerations, this research will focus on students’ purchasing decisions at vending machines. With the final aim of encouraging the selection of healthier options, it will assess the efficacy of different nudge interventions targeting different sensory channels. Additionally, it will explore the implicit cognitive and emotional responses to these interventions, providing insights into the mechanisms underlying (healthier) food decision-making.

The following literature review will first analyze the application of nudge interventions to encourage healthier food choices in educational settings. While most studies focus on school cafeterias, few address vending machines—despite their predominance of unhealthy snack options—highlighting the need for further research in this context (Section *“Nudge and its applications in the food choice domain”*). Next, it will explore how cues from different sensory modalities influence food choices and shape consumers’ cognitions and emotions (Section *“Beyond taste: how sensory inputs shape food choices”*). Finally, it will discuss the appropriate approach for assessing how sensory stimuli and cognitive and emotional responses shape food choices. It will provide an overview of the consumer neuroscience approach and focus on the relevant tools and measures (Section *“The role of consumer neuroscience in the study of food choices”*).

### Literature review

The vending market, now valued at $16.07 billion, is rapidly growing and is forecasted to expand further to $26 billion by 2030 [9]. Vending machines are prevalent in various settings—stations, supermarkets, cinemas, shopping malls, hospitals, workplaces [10], and educational institutions [11]—offering a variety of snacks and beverages in a convenient and accessible way [9]. However, their offerings often include hyperenergetic and sugar-laden options [12], with the proportion of unhealthy foods ranging from 85% to 100%, while the one of unhealthy beverages from 49% to 80% [13,14].

Given this high prevalence of unhealthy options, one question arises: how can healthier choices be effectively promoted in vending machines? In this context, among different strategies and interventions, nudge has emerged as a promising approach to guide consumers toward better dietary decisions.

#### Nudge and its applications in the food choice domain.

As defined by Thaler and Sunstein [15, p. 6], a nudge is *“any aspect of the choice architecture that alters people’s behavior in a predictable way without forbidding any options or significantly changing their economic incentives.”* Different categories of nudges exist [16]. However, when viewed through the lens of libertarian paternalism, they all have the same objective: to empower individuals to make decisions that improve their quality of life and promote their well-being - as well as that of their environment - through strategies that encourage beneficial choices while preserving individual freedom [17]. The contributions arising from the concepts of nudge and libertarian paternalism have significantly enriched behavioral research and the study of choices, impacting several fields such as economics [18], finance [19], public health [20], and psychology [21].

When putting nudge interventions into practice, several factors need to be considered. For instance, excessive choice manipulation can trigger reactance [22], a form of psychological resistance that causes people to reject ideas or actions perceived as external impositions. Indeed, a nudge is most effective when it operates through what Stanovich and West [23] and later Kahneman [24] identified as “System 1” of human decision-making, characterized by its automatic, fast, and low-effort nature.

In addition to nudging, traditional approaches can also be used to encourage healthy choices and improve individual habits. Direct approaches include nutritional education and awareness campaigns to equip individuals with the knowledge and skills to make informed food choices. Indirect approaches, on the other hand, encompass economic incentives, taxation, and government policies, such as financial rewards or penalties, designed to encourage or discourage specific behaviors [25]. However, these strategies can be perceived as intrusive and often encounter resistance. In contrast, nudging initiatives are less invasive and more cost-effective for the entities proposing them [26].

These characteristics have led to the implementation of a variety of nudge strategies to promote healthier eating [27] across different contexts, such as cafeterias, restaurants [28], and supermarkets [29]. To encourage healthier choices, nudge interventions tipically employ novel or personally relevant cues, messages promoting socially normative food selections, and strategies setting healthy options as the default choice [30]. A meta-analysis [31] compared the effectiveness of these different strategies and found that behavioral nudges (e.g., reducing unhealthy foods portion sizes or making healthy options more accessible) were the most effective in reducing unhealthy eating, while cognitive nudges (e.g., providing nutritional information via labels) had a smaller impact.

In educational settings, nudge initiatives often involve adjustments to food presentation and placement, implemented in environments such as cafeterias. For instance, using smaller plates has been shown to reduce portion sizes and calorie intake [32], while placing health-related posters can promote healthier choices [33]. Furthermore, setting the healthier option as default can encourage its selection [34], and strategically rearranging buffets to include fruits and vegetables in a prominent position has been shown to increase their visibility and facilitate their selection [35]. Additionally, placing sugar-sweetened beverages on the lower shelf of refrigerators has been demonstrated to reduce their visibility and discourage their purchase [36].

While nudging has been extensively applied within educational environments in canteens and cafeterias, its implementation in vending machines remains relatively understudied. Examples of nudge interventions in vending machines include [37] and [38]. The first showed that nature-themed posters were more effective than those on other topics—e.g., sports—in promoting healthier snack choices, while the second revealed that dark-colored vending machines led to a preference for caffeine beverages. In contrast, strategies such as highlighting nutritional information or limiting high-calorie products are widely employed [39]. Among these, limiting high-calorie options is the most invasive, yet appears to be the most effective way to promote healthier choices [40].

This limited use of nudge interventions in vending machines highlights the need for further research within this context, especially within educational settings where they can significantly influence students’ dietary habits and well-being.

#### Beyond taste: How sensory inputs shape food choices.

Sensory inputs also have the potential to serve as effective tools for guiding dietary choices, holding promise for their implementation as nudging cues within food environments.

Among sensory-based nudges, olfactory interventions have a unique influence on behavior, producing an immediate and instinctive reaction operating below the level of conscious awareness [41]. Smell can alter our mood and, consequently, our behavior [42] due to the strong overlap between brain regions processing smell and emotions [43]. For this reason, signals from retro- and ortho-nasal olfaction play a key role in food choices [44]. Odors associated with unhealthy foods may prime consumers to make unhealthy food choices [45]. In particular, exposure to sweet odors can influence the likelihood of purchasing sugary foods, while savory odors tend to increase cravings for savory foods [46,47]. Moreover, non-food odors, such as cedarwood or eucalyptus, have also been shown to affect our appetite and food preferences [44,47]. For example, a jasmine scent can significantly diminish cravings for chocolate products [48] while promoting a sense of relaxation that can be related to reduced eating behaviour [49].

Visual cues, such as color and brightness, have also been shown to shape consumers’ expectations of a product’s healthiness [50] and can be strategically used to encourage healthier food choices [51]. Packaging characteristics are often used in this context, with elements such as lighter typefaces and specific colors like green [52] commonly associated with healthier food products [53]. Tonkin and colleagues [54] suggest that presenting a healthy food picture before making food choices can increase the likelihood of selecting healthier options. Similarly, Kemps *et al*. [55] found that presenting a healthy food picture can reduce the subsequent food intake. These types of visual-based interventions are frequently applied in food menu design, where presenting healthy cues separately from other options has been found to enhance healthy food choices [56].

Auditory cues can also influence food consumption frequency, preferences, as well as taste perception [57]. For instance, higher-pitched music has been shown to encourage the selection of healthy options [58]. Conversely, low-volume music promotes relaxation and increases healthy foods sales, while loud music increases arousal and leads to unhealthy food choices [59]. Finally, listening to soundtracks considered “healthy”—i.e., featuring relaxing classical or jazz music—can promote healthier food choices, with visual attention to those options acting as a mediating factor [60].

While these findings illustrate how cues from different sensory modalities can shape food choices - with the potential of fostering healthier habits - research in neuroscience and psychology also highlights the role of multisensory integration in decision-making. The convergence of different but congruent sensory inputs has been shown to enhance attentional focus, emotional engagement, and cognitive efficiency, all of which contribute to more effective behavioral responses. For instance, congruent visual-olfactory stimuli are identified more rapidly than unimodal stimuli, which facilitates object recognition and decision speed [61]. Similarly, visual-olfactory stimulation can more effectively engage neural networks involved in memory and emotional processing compared to unimodal stimulation [62]. These findings provide a theoretical foundation for the idea that combining visual and olfactory nudges may result in more engaging environments, ultimately leading to healthier snack choices. Additionally, insights from different research areas also emphasize that the absence of a multisensory interaction can impair emotional connection and attention [63], further reinforcing the importance of (multi)sensory presence in shaping meaningful choices.

This raises two questions: which of these sensory cues is most effective in nudging towards healthier options? And is combining them indeed more effective? To assess the efficacy of these different cues, it is essential to understand not only which one mostly affects the final decision but also the cognitive and emotional processes that drive this decision, as different cues from different sensory modalities trigger implicit responses that ultimately shape food choices. Indeed, odor exposure has been shown to modulate brain activity [64] in the regions associated with odor processing and recognition [65]. Similarly, visual cues can activate posterior brain areas [66], while auditory stimuli affect oscillatory dynamics in brain regions devoted to sound attention [67] and localization [68]. Assessing and understanding these brain activations is essential as they are related not only to the sensory cues themselves, but also to a variety of cognitive and emotional processes that shape consumers’ choices [69]. In this context, a consumer neuroscience approach can provide useful insights by analyzing the impact of different sensory stimuli on consumers’ implicit cognitive and emotional processes, and could help explain why certain nudge interventions are more effective in shaping behavior.

#### The role of consumer neuroscience in the study of food choices.

Research on consumer behavior has long highlighted the complexity of human decisions, challenging the traditional view of consumers as purely rational. As Knutson and colleagues posit [70], models considering decisions as a rational assessment of costs and benefits fail to consider factors such as emotions, cognitions, social influences, experiences, and contextual cues, which significantly shape consumer preferences and choices. Therefore, to fully understand how food choices are made and how they can be influenced by a nudge intervention, all these variables should be assessed. But how?

Consumer neuroscience addresses this gap by combining insights from neuroscience, psychology, marketing, and economics to enhance our understanding of consumers [71]. The integration of traditional marketing methods with neuroscientific ones, such as electroencephalography (EEG), skin conductance (SC) monitoring, and eye-tracking (ET), aims to better assess these cognitive and emotional processes driving consumer behavior and decision-making processes [72]. Among the various applications of consumer neuroscience—which include the study of stimuli such as packaging, commercials, static advertisements, and websites [73], as well as different consumer experiences [74]—one of the most significant areas of investigation is indeed the one of food choices [75].

In this area, the influence of environmental factors on healthy food choices has been studied through EEG to better understand people’s responses to these cues. For instance, Piper *et al*. [76] found that picture-based warning labels on high-fat foods significantly reduce purchasing intentions by eliciting negative emotions and activating the prefrontal cortex. Indeed, differences in the prefrontal EEG activity can provide insights into consumers’ emotional responses and motivational behavior, through the so-called Approach Withdrawal Index (AWI). AWI measures the activity between left and right prefrontal cortex, with greater left activity associated with approach emotions and behavior, and greater right activity with withdrawal [77,78]. It is one of the most widely used indices, based on the activation of specific cortical regions within distinct EEG frequency bands, to assess consumers’ emotional and cognitive processes during tasks [72], such as food choices. Indeed, in the food choice context, AWI has proven effective in predicting the pleasantness and the selection of food from the moment of cooking [79] and in assessing a greater interest in novel foreign foods compared to familiar ones [80]. AWI has also reliably assessed people’s responses to different sensory cues, making it relevant for the aims of this research. It has shown differences in consumer responses to pleasant vs unpleasant odors [81] and has assessed how smell can improve emotional state and mood [82]. Similarly, it has shown its reliability in capturing people’s emotional reactions to visual cues—such as pictures [83,84]—and auditory cues [85].

Beyond emotional and motivational responses, another key factor influencing food choice is consumer engagement, which not only enhances product value but can also promote healthier choices by fostering consumer loyalty and influencing both intentions and actual purchases [86]. Moreover, it is often linked to the elicitation of positive emotions [86]. Consumer engagement can also be significantly influenced by sensory stimuli, as the integration of different, but congruent, sensory cues can create more immersive and engaging consumer experiences [87]. Engagement is typically measured via EEG with the Beta/Alpha-Theta Ratio (BATR index) [88,89], which could implicitly assess consumers’ attentional and cognitive processing during food selection.

In addition to EEG, other neuroscientific measures can offer insights into consumers’ emotional experiences related to food choice. SC, for example, measures changes in sweating associated with the autonomic nervous system, making it a reliable indicator of arousal, one of the main dimensions of emotional experiences [90]. Heightened SC has been observed when individuals encounter their favorite food or drink [91], proving it as a predictor of food acceptance, stronger than self-reports [92]. While most studies focus on general food preference and choice, SC can also assess emotional reactions to inform strategies that promote healthier eating by aligning food offerings with positive emotional experiences [93]. SC can, therefore, be used not only to understand food preferences but also to provide feedback on individuals’ emotional states in response to specific sensory stimulation. For example, Manzoni *et al*. [49] showed that a state of relaxation can reduce eating. Since relaxation and arousal might represent different emotional regulation strategies, using SC to measure relaxation toward a nudge intervention could indicate an emotional state driveing lighter and healthier food choices.

Finally, along with EEG and SC-based measures, eye-tracking is another useful consumer neuroscience tool: by assessing gaze patterns, fixation duration, and pupil dilation, it can reveal how visual elements capture consumers’ attention and guide their food decisions. For instance, Wang *et al*. [94] showed that fixation on a healthy product can predict its subsequent choice. However, looking at health labels does not consistently result in healthier choices [95]. Furthermore, research indicates that consumers look more at high-calorie than low-calorie foods [96], at selected foods than unselected ones [96], and that they are more willing to pay for foods that have been previously observed by others [97]. Another aspect studied through eye-tracking is the attention given to products based on their placement. It is established that eye-level shelf positioning attracts more attention and increases product selection [98]. Eye-tracking studies have confirmed that eye-level products receive more fixations [99] and that, consequently, placing healthy foods in that position can promote healthier choices [100].

These consumer neuroscience findings contribute to understanding the mechanisms underlying food choice behavior and emphasize the importance of the cognitive and emotional processes affecting these decisions. A consumer neuroscience approach integrating behavioral analysis with EEG, SC, and eye-tracking has the potential to not only facilitate the nudge interventions assessment, but also to contribute to a deeper understanding of the implicit mechanisms influencing food choices, ultimately informing the design of more effective health interventions.

### Research objectives and hypotheses

Based on research highlighting the negative impact of vending machines options on student diets [7,8], the limited use of nudge interventions in this context, and on literature indicating how specific sensory stimuli can facilitate healthier food choices (e.g., visual [37] or olfactory cues [48]), this research aims to explore how sensory stimuli in vending machine settings can nudge students towards healthier snack choices.

Three sensory stimuli will be tested: olfactory (O), visual (V), and a combination of both (VO), compared to a neutral condition (N) (for further details on O, V and VO stimuli, refer to Section *“Experimental nudge conditions”*). Although literature also indicates the role of auditory cues in food choices, we decided to focus on olfactory and visual stimuli, as they can be more easily transferred to real-world settings (while, for instance, the implementation of music in university corridors during lecture breaks may prove ineffective due to excessive external noise).

As sensory stimuli can influence food choice behavior not only explicitly but also implicitly at cognitive and emotional levels [49,82,83], this research goes beyond merely comparing snack choices across nudge conditions: it aims to assess the implicit emotional, cognitive, and attentional impacts of these interventions. For this reason, consumer neuroscience tools will be used. SC will measure emotional arousal, the EEG-based AWI will estimate emotional responses and motivational behavior [78], the EEG-based BATR Index will measure engagement [89], and eye-tracking will assess visual attention [101]. These measures constitute the most popular toolkit in consumer neuroscience, having been adopted in 35%, 10%, and 15% of studies, respectively [102]. These dimensions will be compared across nudge conditions, as well as throughout the decision-making process flow, which has been divided into two distinct phases: the observation (Observation) of the vending machine and the subsequent actual snack choice (Choice).

This two-phase structure is based on decision-making research, which suggests that the initial observation phase strongly shapes preferences [103,104], while the choice phase is associated with higher cognitive activation, emotional arousal [105,106], motivational responses [107] and engagement [108]. Eye-tracking studies also show that early observations can predict later choices [94,96]. However, to fully understand the underlying mechanisms, eye-tracking data should be integrated with neurophysiological measures. By considering both attentional responses (eye-tracking) and neurophysiological reactions (SC and EEG) from the very first moments of product observation, we can gain deeper insights into decision-making processes under nudge conditions.

The following research questions and hypotheses have been developed based on their corresponding levels of analysis (behavioral, neurophysiological, and attentional). Table 1 provides a summary of the different analyses with their respective contribution, measurements, and methods used.

**Table 1:** Current study’s levels of analysis.

Level of analysis	Contribution	Measure	Method
Behavioral	Which type of snack do consumers choose?	Chosen snack type (healthy vs unhealthy)	Choice observation and counting
Neurophysiological	Are consumers cognitively engaged?	Cognitive Engagement (BATR)	Electroencephalography (EEG)
	Are consumers likely to approach the snacks?	Approach Withdrawal Index (AWI)	
	Are consumers feeling relaxed vs aroused?	Arousal	Skin Conductance (SC)
Attentional	How do consumers visually explore healthy snacks?	Time Spenteak Time To First Fixation	Eye-tracking

Summary of the different levels of analysis employed in this study: behavioral, neurophysiological, and attentional, including the corresponding contributions, measures, and methods used for each analysis.

*Behavioral analysis*. This level of analysis aims to evaluate how different nudge interventions influence food choices. Building on literature highlighting the effectiveness of olfactory and visual cues in nudging healthier selection [37,48] and that multisensory effectiveness over unimodal approaches [61,62,109], we hypothesize that:

**H1**: Nudge interventions will lead to a higher selection of healthy snacks compared to N, with VO condition being more effective than V or O in increasing the selection of healthy snacks.

*Neurophysiological analysis*. This level of analysis aims to evaluate consumers’ cognitive and emotional states evoked by the different nudge conditions, assessing physiological arousal via SC, emotional direction and interest via AWI, and engagement via BATR. Building on the literature linking a relaxed state to healthier food choices [49], we hypothesize that:

**H2**: Nudge interventions will result in lower levels of SC compared to N, indicating an increased state of relaxation induced by the nudge, with VO condition resulting in even lower SC compared to V or O.

Since AWI can effectively assess consumers’ response to olfactory and visual stimuli [82,83], and reliably reflect the interest in specific foods [79,80], we hypothesize that:

**H3**: V and O nudge interventions will result in higher AWI compared to N, indicating increased motivation and interest toward the snacks induced by the nudge, with VO condition resulting in even higher AWI compared to V or O.

Given the importance of engagement—measured via BATR—in promoting healthier food choices [86], and considering how it can be influenced by external sensory cues [87], we hypothesize that:

**H4**: V and O nudge interventions will result in higher BATR compared to N, indicating increased cognitive engagement induced by the nudge, with VO condition resulting in even higher BATR compared to V or O.

As decision-making processes are typically associated with heightened physiological arousal [106], motivational responses [107], and cognitive engagement [108], we hypothesize that:

**H5**: The actual snack choice will result in higher SC, AWI, and BATR than simply observing the vending machine.

*Attentional analysis*. This level of analysis aims to assess how different nudge interventions and different snack positioning on the shelves influence attention toward them. Since nudge interventions are expected to increase healthier snacks selection [37,48], we hypothesize that:

**H6**: V and O nudge interventions will lead to increased attention to healthier snacks, compared to N, with VO condition resulting in even higher attention paid to healthier snacks compared to V or O.

Moreover, since both traditional studies [98] and eye-tracking research [99] highlight the advantage of positioning food products on eye-level shelves, we hypothesize that:

**H7**: Positioning healthy snacks at eye level rather than on lower shelves will result in increased attention to them

## Materials and methods

### Participants

A total of 88 students (64 females, 24 males) from IULM University (Milan, Italy), with ages ranging from 19 to 31 years (M = 21.12, SD = 2.16), participated in the study voluntarily. As this study is intended to become an intervention at IULM University, the sample was selected to reflect the institution’s gender proportions (73% female) and was distributed evenly across the different experimental conditions (Gender×Condition Contingency Table: $\chi^{2}$(3) = 6.765, *p* = .08).

The sample size was previously assessed through two sensitivity analyses conducted via G*Power 3.1.9.6 [110]. The “Goodness-of-Fit for Contingency Tables” model (with α = .05, 1-β = .95, *df* = 2, *N* = 88) yielded a minimum detectable effect size of *w* = .419, while the one for the Within-between Experimental Design (with α = .05, 1−β = .95, *N* = 88, *Nmeasures* = 2, ρ = .50, ϵ = 1) yielded an effect size of *f* = .226. The effect sizes, interpreted respectively as “ medium-to-large” and “small-to-medium” [111], are lower than the “more-than-large” median value (*d* = .93) commonly found in recent experimental psychology and neuroscience literature [112].

Eligibility criteria included the absence of food allergies or dietary restrictions (e.g., vegan diet) and non-smoking status to guarantee uniform exposure to nudge cues and to account for potential olfactory impairments in smokers [113]. Participants were informed about the study’s non-invasive nature, voluntary participation, and data anonymity; however, they were not informed about the study’s purpose until the end of it. Additionally, participants were rewarded with a complimentary healthy snack at the end of the experiment, which they had not been informed of to prevent any potential influence on their decisions. Participants recruitment and data collection started on September 23, 2024 and ended on December 20, 2024.

The study was conducted in accordance with the Declaration of Helsinki and was approved by the IRB (approval n. 0073876). All participants provided written informed consent before taking part in the study. The consent forms were signed both by each participant and by a research team member and securely stored in accordance with local Data Protection Regulations.

### Instruments

Our specific research questions and objectives led us to use the following neuroscientific tools: electroencephalography (EEG), skin conductance (SC), and eye-tracking. Please refer to Table 1 for an overview of these tools, their metrics, and their corresponding research purposes.

#### Electroencephalography (EEG).

The EEG was recorded via the NVX-36 device (Medical Computer Systems, Ltd., Moscow, Russia) with 22 Ag/AgCl electrodes on Fp1, Fp2, F7, F3, Fz, F4, F8, T3, C3, Cz, C4, T4, T5, P3, Pz, P4, T6, Po3, Po4, O1, Oz, O2, 2 Ag/AgCl clips on the left and right ear lobes, and 1 Ag/AgCl adhesive patch on the right mastoid (M2). A monopolar montage referenced to M2 was used. The sampling rate was 2 kHz, the vertical resolution 24 bits. Before electrode application, the skin was prepared with scrubbing gel (Nu Prep, Spes Medica, S.r.l.) and conductive cream (Neurgel, Spes Medica, S.r.l.) to keep electrode impedance below 10 kΩ [114]. Signal quality was checked before starting the recording, controlled by the NeoRec software (Medical Computer Systems, Ltd.).

#### Skin conductance (SC).

The SC was acquired via the GSRSens sensor (Medical Computer Systems, Ltd., Moscow, Russia) through 2 Ag/AgCl electrodes on the non-dominant hand index and middle finger. Following international recommendations [115], the conductance was estimated through the constant voltage mode (0.5 V). The GSRSens was connected to one auxiliary input of the NVX-36: the SC signal was, thus, digitized at the same sample rate and vertical resolution as the EEG.

#### Eye-tracking.

The eye-tracking was recorded via the wearable head-mounted Tobii Pro Glasses 3 (Tobii AB, Stockholm, Sweden). Gaze position was tracked at a 100 Hz sample rate with an accuracy of 0.6° according to the dark-pupil mode [116] through 16 infrared LEDs and 4 cameras. The recording was controlled by the Glasses 3 Controller app (Tobii AB).

#### Instrumentation fitting and data synchronization.

As participants were required to stand, move, and interact with the vending machine and its products, the NVX system and the eye-tracking recording unit were placed in a backpack worn by the participant.

A trigger-based approach was used for offline data synchronization. At the beginning of the recordings, the Tobii Glasses 3 generated a TTL pattern recorded from the NVX-36 through the ESB, a custom-made EEG Synchronization Box [117].

### Experimental procedure

**Fig 1:**
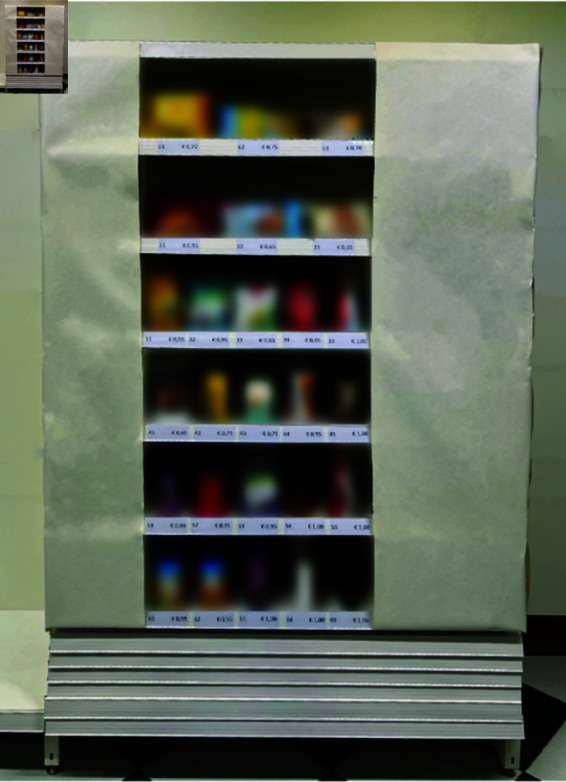
Vending machine replica used in the lab experiment. The vending machine replica setup mirrors the actual vending machine on our university campus, ensuring consistency in dimensions, product quantity, positioning, and pricing. For copyright reasons, the products’ details have been blurred.

The experiment took place at the IULM University NeuroRetail Lab, where a section of shelves was arranged to replicate a vending machine (Fig 1). Specifically, we recreated a real vending machine from our university campus, located near the lecture halls most frequently used by students. The replication ensured consistency in vending machine dimensions, product quantity, arrangement, and positioning. Product selection reflected that of our campus vending machine, including sweet (e.g., cakes, brownies, chocolate bars) and savory snacks (e.g., crackers, breadsticks, chips), and beverages (juices). Water was excluded. Prices were maintained as they are critical for product evaluation [118], and their removal would have compromised the study’s ecological validity.

Participants arrived and sat in front of a 23.8^′′^ LCD monitor at a distance of 60 cm. First, EEG and SC sensors were positioned; then, participants wore eye-tracking glasses, adjusted to ensure a comfortable fit and clear vision. Participants were instructed to stay as still as possible to ensure eye-tracking accuracy and reduce SC and EEG artifacts. Once the preparation was completed, the eye-tracking was calibrated. Participants then underwent two relaxation phases to record baselines: the first (EYC), with eyes closed, lasted 60s, and the second (BSL), with eyes open while observing a black screen with a white dot in the center, lasted 120s. Finally, to fully engage participants in the task, establish a consistent scenario across all participants, and simulate a realistic purchasing experience at university vending machines, the researcher provided a tailored briefing: *“Imagine a typical day at university. You’re in the IULM1 building. You’ve already attended a few classes, and now it’s mid-afternoon, around 4:30 p.m. You’re starting to feel slightly tired and need to take a short mental break. As you walk through the university halls, still thinking about the class you just finished—or the one coming up—your eyes land on a vending machine. It’s one you’ve passed many times before, filled with the usual snacks and drinks. You walk toward it.”*

Participants were thus guided into the experimental room. Here, the illumination was controlled, with only the area surrounding the vending machine illuminated, directing attention to it while keeping the rest of the room dimly lit. Following the literature on the phases of the decision-making process [103–108], participants were then asked to:

– first, stand in front of the vending machine to observe it and its products for 10s, based on the literature on product shelf observation [119,120], to compare observation behaviors across conditions in the attentional level analysis (Observation phase);
– then, following the researcher’s instructions, they indicated the product they would have selected (Choice phase). Time restrictions were not implemented in this phase.

Participants then completed a final questionnaire to control for variables such as BMI, hunger level, and general health interest, ensuring they were consistent across conditions and did not impact the nudge intervention. BMI was assessed with participants’ weight and height (*BMI* = *weight*/*height*^2^). Hunger level was assessed with a single item, and general health interest with the General Health Interest dimension of the Attitudes Towards Health and Taste Scale - Italian version [121]. Both hunger level and general health interest items had to be answered on a 1 to 6 scale chosen to discourage neutral responses by omitting the middle option [122].

The entire experimental procedure had a total duration of 45^′^ per participant.

#### Experimental nudge conditions.

Participants were randomly assigned across 4 experimental conditions, each corresponding to a different nudge intervention.

– *Neutral (N)*: Participants were presented with the vending machine replica without any nudge.
– *Visual (V)*: Drawing on literature showing that visual cues—i.e., nature-themed images—are effective in promoting healthier choices [37], a nature-themed image was employed as visual nudge. The image, consistent with the original study’s specifications regarding the object, colors, contrast, and brightness, measured 50×70 cm and was positioned at eye level on the left side of the vending machine (S1 Fig).
– *Olfactory (O)*: Based on literature indicating the efficacy of olfactory cues in promoting healthier eating, such as the finding that jasmine scent reduces chocolate cravings [48], participants were presented with the vending machine accompanied by a jasmine scent. A jasmine essential oil was diffused in the room via an aroma diffuser, activated 30 minutes before participants’ arrival, set on a continuous mode to ensure accurate scent diffusion, and positioned out of sight to avoid participants’ awareness of the intervention. The essential oil and the aroma diffuser are shown in S2 Fig.
– *Visual + Olfactory (VO)*: Participants were presented with the vending machine replica accompanied by both visual and olfactory nudges. Given that both visual and olfactory cues can effectively promote healthier choices, this condition was implemented as we expect an incremental effect from combining these two interventions.

Participants assigned to the O and VO conditions completed the experiment on different days from those in the V condition, ensuring that the V condition, which did not involve any scent, remained completely unaffected by any odor that could have remained in the experimental room.

#### Snack classification.

The classification of the vending machine snacks as Healthy or Unhealthy was based on a questionnaire, as snack’s perceived healthiness depends more on packaging communication than its actual nutritional content. In the context of vending machines, consumers are indeed unable to directly access the nutritional information of products and are therefore required to rely on packaging cues to assess their perceived healthiness [123,124]. This perception-based categorization of snacks through questionnaires was in fact adopted in other similar studies [37].

Snack’s perceived healthiness was assessed by the Health dimension from the Food Choice Questionnaire [125]. It comprised 6 items, first translated into the target language (Italian) and then back-translated to the original language to ensure consistency. Participants were presented with images of each snack available in the vending machine (N = 26) and asked to indicate their agreement with each item on a Likert scale (1 = strongly disagree, 6 = strongly agree). To prevent any presentation order bias, product images and questionnaire items were randomly presented. The questionnaire was distributed online and completed by a total of 131 voluntary participants (68% females, 31% males, 1% nonbinary), aged 19–64 years (M = 26.24, SD = 6.46). Given that students from our university were recruited as participants for the aforementioned experimental study, they were excluded from completing this questionnaire to preclude any potential influence on their following product selection. Responses from participants with food allergies, intolerance, or following specific diets (e.g., vegan) were also excluded, leading to a final sample of 100 respondents (65% females, 35% males) aged 20–64 (M = 26.51, SD = 7).

Internal reliability analysis (S1 Table) showed excellent reliability within the Health dimension (α > .9), meeting the criterion of exceeding .70 [126].

Product perceived health scores were computed (S2 Table) and, following similar studies [127], dichotomized into two categories based on a median split: Healthy (scoring above median) and Unhealthy products (scoring below median). Snacks’ perceived health scores significantly differed between Healthy (M = 12.74, SD = 3.857) and Unhealthy (M = 7.877, SD = .372) snacks (*t*(24) = –4.526, *p* < .001).

### Data processing

EEG and SC signals were processed using Matlab (The Mathworks, Inc., Natick, MA, USA), according to a previously adopted pipeline [128].

The EEG processing was carried out with the EEGLab toolbox [129]. The EEG signal was re-referenced to the linked earlobes, re-sampled to 512 Hz and filtered with a band-pass (0.1–30 Hz) and notch (50 and 100 Hz) filter. Non-stationary artifacts (e.g., movements and external noise) were corrected through the Artefact Subspace Reconstruction [130], while stereotypical artifacts (e.g., blinking and muscular noise) were corrected through the Independent Component Analysis based on SOBI algorithm [131] and ICLabel classifier [132]. Finally, a re-reference to the Current Source Density was applied to increase the spatial resolution at the sensor level [133]. The cleaned EEG was aligned to the starting TTL pattern and epoched according to task onset and duration. For each subject, the Individual Alpha Frequency (IAF)—the center of gravity of the Power Spectral Density (PSD) within the extended alpha range (7.5–12.5 Hz) [134]—was computed. In the IAF calculation, the mean PSD averaged across all the occipital channels was considered. The PSDs were computed on the EYC data according to Welch’s method with a 1s-long Hamming window and 50% overlap. The IAF defined the individual alpha band α=[IAF−2;IAF+2] [135].

The SC was band-pass filtered (0.001—0.35 Hz) and down-sampled to 32Hz. Then, artifactual points were identified as those exceeding three thresholds (minimum amplitude of 0.05 μS, maximum amplitude of 60 μS, rate of change of ±8 μS/s) and replaced by a linear interpolation [136]. SC signal was finally aligned to the starting TTL pattern and epoched according to task onset and duration.

From the EEG signal, AWI was computed as the difference of α powers between right and left prefrontal channels, while BATR was calculated as the ratio between β powers and the sum of α and θ powers across all EEG channels. The calculation was based on the Short-Time Fourier Transform with a 1s-long Hamming window and 50% overlapping, as it shows better performances compared to the filtering approach [137].

To reduce inter-subject differences between the neurophysiological signals, AWI, BATR and SC were z-scored according to the mean and standard deviation values within the BSL epoch [138]. Then, they were temporally averaged to obtain a condensed stimulus-related value [139].

Eye-tracking data was processed and analyzed using Tobii Pro Lab (Tobii AB), with areas of interest being created on target stimuli (healthy snacks) to extract the following measures:

– *Time Spent (TS)*, the total duration of fixation (ms) on the Healthy snacks; it provides insights into the overall attention allocated to that area.
– *Time to First Fixation (TTFF)*, the temporal difference (ms) between stimulus presentation (Healthy snacks) and the first fixation on it; it offers insights into initial attentional capture.

Examples of the application of these eye-tracking measures in consumer neuroscience studies can be found in the works of Russo and colleagues [128,140].

### Statistical analysis

Statistical analyses were performed via R Statistical Software, v4.1.2 [141], using the contingecytables, v3.0.1 [142] package for the behavioral level analysis, and the lme4, v1.1-35.5 [143], emmeans, v1.10.5 [144], ggplot2, v3.5.1 [145], and afex, v1.4-1 [146] packages for the neurophysiological and attentional level analyses.

The homogeneity of the sample regarding BMI, Hunger level, and General Health Interest across conditions was evaluated through a 1-way ANOVA, considering as factors the experimental condition (4 levels: N, V, O, VO).

For the behavioral level analysis, to assess the differences in the product choice due to the experimental conditions, three 2×2 contingency tables (i.e., [N vs V] × [Healthy vs. Unhealthy], [N vs O] × [Healthy vs. Unhealthy], [N vs VO] × [Healthy vs. Unhealthy]) were computed and chi-squared tests were applied.

For the neurophysiological level analysis, for each y-neurophysiological index (AWI, BATR, and SC), 3 linear mixed models (LMMs) were tested, including Condition (N, V, O, VO) and Phase (Choice, Observation) as fixed effects and subjects as random intercept effect. To choose models, we performed model fit comparisons using AIC, BIC, through the R-base function anova [147,148] setting y ~ Condition*Phase + (1| subjects) as the full model. Other 3 models were tested: no interaction between Condition and Phase, only Condition, and the null model. Model fit comparison results are reported in the S1 Appendix (Section 1). Planned contrast used the R-standard dummy code with N for Condition and Choice for Phase as reference levels. For the attentional level analysis, two LMMs were tested (one for TS and one for TTFF), including Condition (N, V, O, VO) and product Shelf positioning (Above, Below) as fixed effects and subjects as random intercept effect. Regarding snack positioning on shelves, given the presence of 6 shelves in the vending machine, snacks positioned on the 3 shelves above were classified as “Above,” while snacks positioned on the 3 shelves below were classified as “Below.” For both TS and TTFF, model fit comparisons were performed to choose models, using AIC, BIC, and $\chi^{2}$ through the R-base function ANOVA [147,148] and setting y ~ Condition*Shelf + (1| subjects) as the full model. For both measures, 4 other models were tested: no interaction between Condition and Shelf, only Condition, only Shelf, and the null model. Model fit comparison results are reported in the S1 Appendix (Section 2). Planned contrast used the R-standard dummy code with N for Condition and Above for Shelf as reference levels. For all the LMMs implemented for neurophysiological and attentional level analyses, outlier removal, assumptions of linearity, homoscedasticity, normality of the residuals, and collinearity (VIF) were graphically assessed using the R-based performance package, v.0.13.0 [149]. In cases where collinearity was detected, it was evaluated whether it was structural by examining the type (fixed effect vs interaction), condition number (k), the stability of the design matrix, and the reliability of standard errors of the model estimates [150]. Moderate deviations from residual normality were first evaluated using simulation-based QQ plots with Monte Carlo-generated envelopes, also testing logarithmic transformation models to determine whether such violations affected the overall interpretation of the model, including the direction and significance of the effects [151]. Post hoc tests on estimated marginal means were corrected using Holm’s method. No convergence issues emerged during the models’ running for estimating parameters.

## Results

The groups exposed to the different nudge conditions resulted balanced in terms of BMI (*F*(3,83) = .092, *p* = .965, $\eta^{2}$ = .003), Hunger level (*F*(3,83) = 1.075, *p*= .364, $\eta^{2}$ = .037) and General Health Interest (*F*(3,83) = .764, *p* = .518, $\eta^{2}$ = .027).

### Behavioral level

Chi-squared tests indicated an association between the O condition ($\chi^{2}$(1) = 4.125, *p* = .042, ϕ = .306) and the V condition ($\chi^{2}$(1) = 5.350, *p* = .021, ϕ = .349) and snack choice (Table 2). While in the N condition, 14% of the selected snacks were healthy, in V and O conditions, 45% and 41% of the chosen snacks, respectively, were healthy. In contrast, in the combined condition (VO), only 36% of the chosen snacks were healthy. While this represents an increase in healthy snack choices compared to the N condition, it is not statistically significant ($\chi^{2}$(1) = 3.030, *p* = .082, ϕ = .262).

**Table 2:** Behavioral results.

lightgray Contingency Table 1, N – VO					lightgray Contingency Table 2, N – O					lightgray Contingency Table 3, N – V			
	Snack category					Snack category					Snack category		
Condition	Healthy	Unhealthy	Total		Condition	Healthy	Unhealthy	Total		Condition	Healthy	Unhealthy	Total
N	3	19	22		N	3	19	22		N	3	19	22
VO	8	14	22		O	9	13	22		V	10	12	22
Total	11	33	44		Total	12	32	44		Total	13	31	44
**Chi-squared tests**					**Chi-squared tests**					**Chi-squared tests**			
	value	df	p			value	df	p			value	df	p
$\chi^{2}$	3.030	1	.082		$\chi^{2}$	4.125	1	.042*		$\chi^{2}$	5.350	1	.021*
N	44				N	44				N	44		

Contingency tables comparing the distribution of snack category choices (Healthy vs. Unhealthy) across three experimental conditions: N, VO, O, V. Chi-squared tests were conducted to evaluate the association between condition and snack choice. Significant associations are highlighted with *.

### Neurophysiological level

Table 3 reports the descriptive statistics (Mean and SD) of the three neurophysiological measures, namely AWI, BATR and SC.

**Table 3:** AWI, BATR and SC descriptive statistics.

Condition	Phase	AWI		BATR		SC	
		Mean	SD	Mean	SD	Mean	SD
N	Choice	0,328	0,657	0,799	1,369	19,786	15,406
	Observation	–0,170	0,439	0,553	2,038	17,452	13,190
O	Choice	–0,060	0,528	0,818	1,995	7,540	4,467
	Observation	–0,119	0,611	0,337	1,731	6,886	4,328
V	Choice	0,349	0,771	1,163	1,660	10,009	11,627
	Observation	0,081	0,416	0,197	2,018	12,246	14,040
VO	Choice	–0,287	0,728	0,953	1,800	7,491	8,944
	Observation	–0,040	0,717	0,202	2,172	6,982	8,436

Descriptive statistics (Mean and SD) of Neurophysiological Indexes (AWI, BATR and SC) for Condition (N, O, V and VO) and Phase (Choice, Observation). Note that AWI stands for Approach Withdrawal Index (neurophysiological measure of emotional direction and interest); BATR stands for Beta-Alpha Plus Theta Ratio (neurophysiological measure of engagement); SC stands for Skin Conductance (neurophysiological measure of arousal).

**Fig 2:**
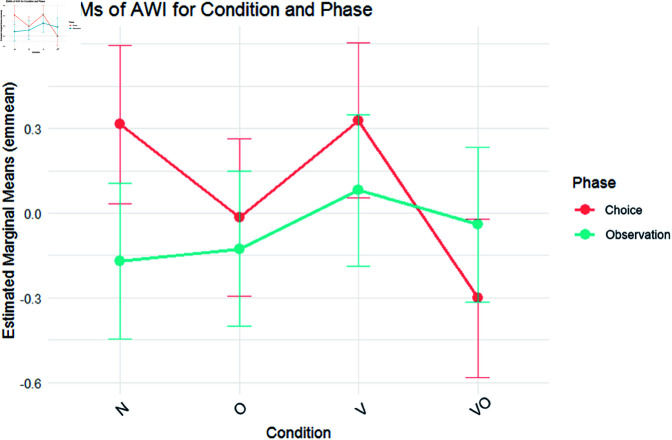
AWI EMMs. EMMs of AWI on phase (choice, observation) and condition (N, O, V, VO). Bars highlight a 95% confidence interval.

The model tested for AWI was the full model, including Condition and Phase and their interaction (Fig 2). Planned contrast in Condition highlighted a significant difference between the reference level and the VO condition, which showed lower AWI (β = –.617, *SE* = .200, *t* = –3.076, *p*<.01). Observation showed lower AWI than the reference level (β = –.485, *SE* = .15, *t* = – 3.244, *p*<.01). However, a significant interaction was found between the VS condition and Observation. The condition VO seems to increase the AWI of the Observation phase if compared to the Choice phase (β = .746, *SE* = .211, *t* = 3.530, *p*<.001). Post hoc tests on estimated marginal means were performed on the interaction between Condition and Phase. Results confirmed the significant increase in AWI of the Choice phase compared to the observational phase only in the N condition (Mean Diff. = .485, *SE* = .150, *t* = 3.243, *p* = .036) but did not show any other significant difference. Random effect analysis showed a dispersion around the intercept of SD_Intercept_ = .413 with an unexplained residual variance SD_Intercept_ = .464. This pattern indicates that while AWI responses were substantially shaped by experimental conditions, a considerable portion of variance was attributable to individual differences in baseline neurophysiological responsiveness.

**Fig 3:**
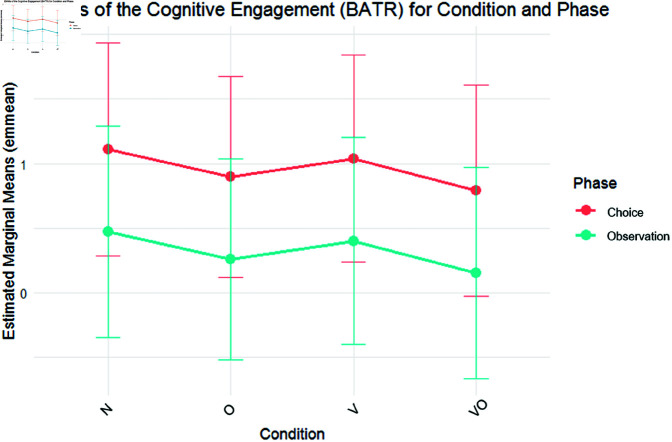
BATR EMMs. EMMs of BATR on phase (choice, observation) and condition (N, O, V, VO). Bars highlight a 95% confidence interval.

The model tested for BATR included Condition and Phase without interaction. Results (Fig 3) showed that neither planned contrast nor post hoc on Condition showed any significant difference. However, the Phase fixed effect showed a decrease of BATR in the Observation phase compared to the Choice (β = –.637, *SE* = .149, *t* = –4.257, *p*<.01). No additional significant differences were provided with post hoc tests. The random standard deviation around the intercept was SD_Intercept_ = 1.517 with a residual SD_Intercept_ = .648. This indicates that BATR levels varied widely between individuals regardless of the experimental condition, suggesting, as expected, high inter-individual variability.

**Fig 4:**
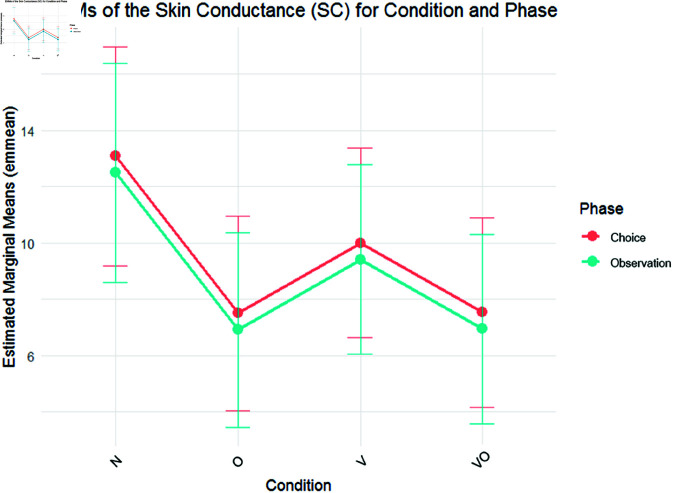
SC EMMs. EMMs of SC on phase (choice, observation) and condition (N, O, V, VO). Bars highlight a 95% confidence interval.

The model tested for SC included both Condition and Phase without interaction. Planned contrast showed a significant decrease of both O (β = –5.560, *SE* = 2.579, *t* = –2.156, *p* = .036) and VO (β = –5.583, *SE* = 2.608, *t* = –2.141, *p* = .035) condition if compared to the reference level. No significant contrast with the V condition was found. The Observation phase showed significantly lower SC if compared to the reference level (β = –.590, *SE* = .2476, *t* = –2.384, *p* = .020). Post hoc tests did not show any additional significance (Fig 4). The random standard deviation around the intercept was SD_Intercept_ = 7.473 with a residual SD_Intercept_ = 1.455. Again, these results highlight strong between-subject variability in baseline skin conductance levels, suggesting that physiological arousal differed substantially across participants.

### Attentional level

**Fig 5:**
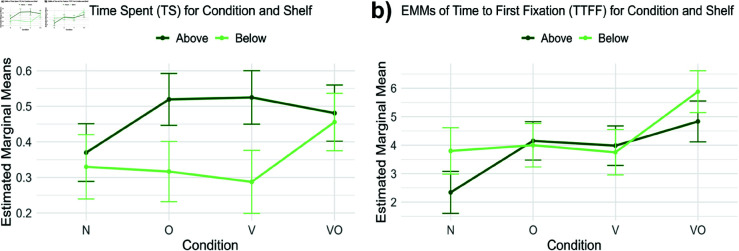
Eye-tracking metrics EMMs. EMMs of time spent, TS (**a**) and time to first fixation, TTFF (**b**) on Condition (N, O, V, VO) and healthy snacks positioning in the vending machine shelves (above, below). Bars highlight a 95% confidence interval.

The model tested for TS was the full model, including the interaction between Condition and Shelf. Planned contrast on Condition showed a significant increase of TS on healthy snacks in O (β = .150, *SE* = .055, *t* = 2.707, *p*<.01) and V conditions (β = .155, *SE* = .056, *t* = 2.769, *p*<.01), compared to N condition. No significant difference between the reference level and VO condition was found. Shelf position did not show a significant difference between Above and Below, but significant interactions were found with specific conditions. In particular, placing products on the Below shelves significantly decreases the TS on healthy snacks in both O (β = –.162, *SE* = .082, *t* = –1.964, *p*<.05) and V (β = –.197, *SE* = .084, *t* = –2.339, *p*<.05) conditions, but no significant interaction was found between Shelf and the VO condition. The dispersion around the intercept was small, with a value of only SDIntercept = .027 and a residual standard deviation SDResidual = .387. This pattern indicates that between-subject variability in baseline Time Spent was negligible and that most of the variance originated within subjects across trials. Such a structure suggests that engagement was more strongly shaped by experimental conditions than by stable individual differences. Post hoc tests on EMMs (shown in Fig 5a) confirmed significantly higher values of TS on healthy snacks in the Above shelves if compared to the Below ones for both O (Mean Diff. = .203, *SE* = .056, *t* = 3.614, *p*<.01) and V (Mean Diff. = .237, *SE* = .058, *t* = 4.065, *p*<.01) conditions. No significant results related to shelf levels were found for N and VO conditions.

The model tested for TTFF was the full model, including the interaction between Condition and Shelf. Planned contrasts on Condition showed an increase in TTFF on healthy snacks in O (β = 1.809, *SE* = .506, *t* = 3.572, *p*<.001), V (β = 1.641, *SE* = .513, *t* = 3.200, *p*<.01), and VO (β = 2.491, *SE* = .522, *t* = 4.772, *p*<.001) conditions compared to N condition when products are in the Above shelves. Generally, placing healthy snacks on the Below shelves significantly increases their TTFF (β = 1.456, *SE* = .503, *t* = 2.892, *p*<.01) in the N condition, but significant negative interactions were found for both O (β = –1.610, *SE* = .679, *t* = –2.369, *p*<.05) and V (β = –1.690, *SE* = .693, *t* = –2.438, *p*<.05) conditions. A negative interaction was also observed in the VO condition, but it was not significant. Post hoc tests on EMMs (Fig 5b) confirmed an almost significant decrease of TTFF on healthy snacks on the Below shelves compared to the Above ones in the N (Mean Diff. = –1.456, *SE* = 0.505, *t* = –2.887, *p* = 0.072), V, and O condition, but not in VO. The dispersion around the intercept was almost one second SD_Intercept_ = 0.797 with a residual standard deviation of SD_Residual_ = 3.132. This indicates moderate between-subject variability in baseline TTFF, with a larger share of variance stemming from within-subject fluctuations across trials.

## Discussion

Research to date highlights the multifaceted nature of nudges and their potential in promoting healthier food choices. However, most nudge interventions focus on visual aspects of the environment (playing with colors, posters, and positioning), overlooking other sensory inputs such as smells. Additionally, they are typically implemented in settings such as cafeterias, with less attention paid to an important context where people frequently purchase unhealthy food: vending machines.

The objective of our research was to investigate the impact of different nudges targeting different sensory channels on students’ snack selections at university vending machines. Our aim was twofold: first, to evaluate the efficacy of the nudge intervention; second, to elucidate and assess the cognitive, emotional, and attentional processes that shape food choices. Three nudge conditions were tested: a Visual cue (a nature-themed image, V), an Olfactory cue (a jasmine scent, O), and a combination of both (VO), and compared to a no intervention neutral condition (N). Along with behavioral analysis, consumer neuroscience tools (EEG, SC and eye-tracking) were implemented to assess the emotional, cognitive, and attentional correlates of these conditions, and throughout the snack decision process (Observation vs. Choice phase). The results revealed interesting differences shaped by the nudge interventions, with effects varying across behavioral, neurophysiological, and attentional levels of analysis.

### Behavioral level: single cues enhance healthy selection, combined cues fall short

The behavioral comparison of snack choices across experimental conditions revealed a significant increase in healthy snack choices in both V and O conditions. These findings are consistent with prior research, which has shown how a nature-themed image [37] or a jasmine scent [48] could facilitate healthier food choices, and partially confirm the first hypothesis (H1).

In contrast, in the combined condition (VO), the selection of healthy snacks was not significantly higher than in the neutral one, suggesting that the simultaneous use of two different nudges may not be as effective as hypothesized. This lack of effectiveness could potentially be attributed to a cognitive load arising from excessive sensory stimulation and from the use of two nudges conveying different messages: on the one side, the intense jasmine scent and on the other side, the image of a tree, which may result as incongruent [152,153]. This idea has already been explored in previous research, which has primarily focused on the interaction of nudges within the same sensory modality[154–157], but with inconclusive findings that highlight the need for further research into this effect. Another potential reason for the reduced effectiveness of multiple sensory cues is a lack of synergy, where different sensory stimuli may conflict rather than complement each other. For instance, olfactory stimuli can alter visual perception, even influencing brain activity in the visual cortex [158]. In this case, instead of enhancing the visual cue, the added scent may have disrupted its effect. This aligns with research showing that cross-modal conflicts can arise between visual and olfactory stimuli, with scent sometimes exerting a dominant influence over visual processing [159].

### Neurophysiological level: Effective nudge interventions induce relaxation and approach behavior

The behavioral insights are supported by the neurophysiological data from SC and EEG.

The SC, reflecting physiological emotional arousal, was lower in the nudge conditions compared to the neutral one, during both Observation and Choice. Significant differences were observed between the O and VO—but not V—conditions compared to the neutral one, partially confirming H2. This is consistent with previous studies showing how olfactory cues can influence arousal levels and decision-making processes [48]. Indeed, the olfactory system’s direct connections to areas such as the amygdala and hypothalamus [160] may trigger rapid emotional responses that significantly affect behavior and choices [161]. Manzoni and colleagues [49] further demonstrated that a relaxed state is associated with better dietary control. Thus, olfactory stimuli promoting relaxation—as shown by lower SC—may potentially encourage healthier food choices. Relaxation can indeed reduce stress and anxiety—factors that contribute to unhealthy eating habits—finally improving healthy eating in specific conditions [162,163]. So, even if its direct impact on reducing energy intake or cravings may indeed be limited, the sense of relaxation could still support strategies for promoting healthier food choices. Moreover, contrary to H2, the combined condition did not lead to lower SC than the single nudge conditions. This is consistent with the behavioral data, where the combined intervention did not outperform the single nudges, suggesting that the combined condition may not be able to induce greater relaxation and may instead increase cognitive load. Finally, the Choice phase evoked higher SC than the Observation phase, likely due to the action of making a choice, a moment that typically induces greater arousal [106].

Regarding EEG, AWI measured participants’ emotions and motivational behavior [78] between conditions and phases. Higher AWI values were generally higher during Choice than Observation, partially disconfirming H5. However, a peculiar dynamic emerged in the combined condition, characterized by a significant AWI decrease during Choice. This could be because, in line with the previous analyses identifying the Choice phase as the most activating [106], the greater cognitive fatigue induced by the combined intervention’s multisensory stimulation may lead the subjects to withdraw from the task [164], as reflected in the lower AWI values. This is also consistent with prior research highlighting that different sensory modalities do not always work in harmony [158], and can instead compete, particularly for attentional resources [165]. In our case, this may have led to cognitive disengagement, causing participants to withdraw from the presented task and stimuli. Finally, the EEG-based measure of engagement (BATR) showed no significant differences between conditions. However, it was higher during the Choice phase than the Observation phase, supporting H5. This finding aligns with previous research indicating that the actual product choice elicits greater engagement compared to simple observation [166] and is consistent with SC data, further reinforcing the idea that the act of choosing a product requires higher cognitive and emotional activation. Thus, H3 and H4 are not confirmed, while H5 is only partially confirmed.

### Attentional level: Combining cues distracts from healthy foods, while eye-level positioning enhances their visibility

Our eye-tracking analysis confirmed the effect of shelf positioning on visual attention: placing healthy snacks below eye level reduces their visibility, leading to less time spent observing them, which supports H7. This finding aligns with prior research showing that products positioned on top shelves attract more attention and are more likely to be chosen [167], as consumers typically evaluate only a limited subset of options [168]. This insight is particularly relevant for vending machine design, where the strategic placement of healthy products at eye level [169] can be used to promote healthier choices.

While acknowledging that consumer attention and choice can be shaped by situational factors like time pressure [170] and contextual cues designed to capture attention or modify perceived value—e.g., health labels [171], traffic light systems [172], or pricing strategies [10]—our findings reveal that the general recommendation to position target snacks at eye level is influenced by the nudge conditions, partially confirming H6. While V and O conditions increased attention toward healthy snacks placed on top shelves, VO condition did not, resembling the N condition’s exploratory patterns. This aligns with our behavioral and neurophysiological findings indicating that the VO condition may be less effective in promoting healthy food choices, likely due to the cognitive load or cross-modal competition for attentional resources [165], which may divert people’s attention away from healthier snacks [173] rather than enhancing their salience.

### Theoretical and practical implications

*Theoretically,* this research contributes to the literature on food choice architecture and emphasizes the need for interventions targeting single, specific sensory cues to promote healthier behaviors. An important contribution of this study lies in its neurophysiological insights into the cognitive and emotional dynamics of food choices, and into how they can be shaped by sensory cues. While prior research has focused primarily on the behavioral aspects of food choice architecture, our study provides a deeper understanding of the underlying neurophysiological mechanisms. The findings suggest that cognitive load, often caused by excessive (multi)sensory stimulation or complex decision environments, can hinder healthier choices. Conversely, states of relaxation facilitated for instance by a specific scent could promote more deliberate and healthy decisions, underscoring the importance of designing environments that reduce cognitive load while promoting calm and focused decisions.

*Practically,* vending machine operators and policymakers can apply these insights to design more effective interventions. However, our findings, for example, highlight that the effectiveness of eye level placement is not uniform across all conditions; its ability to capture attention can be significantly modulated by the surrounding sensory context, such as the specific nudges employed in the environment. This implies that optimal design involves not only placing healthy items centrally but also ensuring they remain salient amidst other potentially competing stimuli. Indeed, sensory stimuli, such as specific scents, have long been strategically employed in physical environments to influence the shopping environment and consumer behavior. For instance, a pleasant scent can enhance store appeal and encourage positive purchasing behaviors, particularly when the scent aligns with the store and its products [174]. Supermarkets, for example, use targeted scents to promote specific products [175], while brands like Subway diffuse the aroma of freshly baked bread to boost sandwich sales [176]; this is why major brands such as Apple, Samsung, and Pandora have developed their own signature fragrances [176]. Our insights can be applied in a similar way in field settings, not merely to increase sales, but to guide consumers toward healthier choices.

Nudging young people toward healthy food can affect their lifestyle in the long term. By preventing weight gain in the first university years, it would be possible to prevent, monitor, and reduce obesity among the future adult population. This could also have important consequences on government issues: healthy populations mean less public health spending, with the possibility of investing in the country’s growth.

### Limits and further research

While our study provides worthwhile insights into the application of sensory stimuli to nudge healthier food choices in vending machine settings, some limitations should be acknowledged. First, the research was conducted in a controlled laboratory setting, which may not fully reflect real-world vending machine usage, limiting its ecological validity. In a real situation, additional contextual elements may modulate the impact of nudge interventions. For example, time scarcity-the perception that time is limited—often shapes food decisions, leading people to rely on their habits or heuristic-based choices [177,178]. In addition, peer influence—the impact of friends or affiliates on individual behavior [179]—can alter food selection, especially in young adults who tend to conform more to social norms [180]. Furthermore, neurophysiological measures are not the only method to investigate the mechanisms underlying food choices. These could also be explored from a qualitative perspective through interviews or focus groups, which may provide complementary insights into participants’ conscious motivations and subjective experiences. Another critical factor is the absence of real money: in the present study, participants selected products without any tangible monetary implications. Although this decision was made to avoid price-driven selection—such as choosing the most expensive items to maximize personal gain [181]—it also eliminates the cost-benefits evaluation process, not fully reflecting real choices when facing financial constraints [182,183]. Future research should address these limitations by evaluating product choices under even more realistic purchasing conditions and replicating the study in natural field settings, such as real university vending machines. Moreover, the study focused specifically on visual and olfactory cues to understand how sensory channels can influence behavior in non-intrusive ways, albeit at the expense of other nudging strategies, such as social proof and framing effects [184,185]. Future research could benefit from a more comprehensive and comparative approach, integrating sensory nudges with cognitive and social mechanisms. This would help identify synergistic effects and optimize the effectiveness of health-promoting interventions.

The long-term effectiveness of these interventions on food choice should also be investigated, examining their impact on dietary habits beyond immediate selections. As long-lasting changes in outcomes strengthen the case for using nudges as a policy tool, further research is needed on the durability of their effects [186]. In the domain of healthy eating, this includes evaluating the role of stable modifications in the food environment and their contribution to the long-term efficacy of nudges [187]. Furthermore, it is important to investigate the influence of repeated exposure to nudges, as their frequency and duration may moderate their long-term impact [188]. Researchers should also consider the extent to which nudges can create routines that become behavioral habits [189]; however, this can be difficult, as new routines are not easy to stabilize due to competing demands on the individual’s time and attention as well as with the physical and cultural context in which these behaviors are rooted [189,190].

A larger sample size could enhance the robustness of the findings, while a more detailed understanding of participants’ motivations and emotional states could inform better-targeted interventions, given their importance in eating behavior [191,192]. Finally, examining the impact of nudges in a variety of settings—e.g., workplace cafeterias or other public spaces—could enhance our understanding of their broader applicability and effectiveness in promoting healthier food choices. In doing so, it would be important to account for differences in demographics, lifestyle, and eating habits, as these factors may influence how nudges are perceived and acted upon in non-academic contexts.

## Conclusions

The steady rise in global obesity emphasizes the urgent need for effective policies to promote healthier diets, especially among younger generations. In this context, vending machines, common in educational institutions and several other settings, deserve special attention. Although they often contribute to unhealthy eating habits [8], this paper shows how, under specific conditions, vending machines encourage healthier choices.

Our findings shed light on how sensory nudges shape consumer decisions and food selection. While single nudges (olfactory or visual) effectively increase healthy snack choices, their combined use appears less effective. Indeed, cognitive load emerges as a critical factor in reducing the impact of nudge interventions and in influencing the choice of junk over healthy food. Thus, to enhance nudge effectiveness, it is essential to minimize it and create a consistent and functional food choice architecture. Additionally, strategic snack placement at eye level boosts visibility and selection.

The use of consumer neuroscience tools in this research enhanced the existing literature on food choice by offering a more direct approach to consumers’ emotional and cognitive responses that drive food choice and can be triggered by specific nudge interventions.

Overall, our results reinforce the idea that well-designed nudges can effectively encourage healthier behaviors, and emphasize the importance of creating a “low-cognitive-load” food choice environment—in line with the concept of “System 1” [23,24], where healthy options are easily identifiable and accessible. Future research should further explore the role of sensory integration in nudge interventions, particularly through in-field experiments, to guide a more effective design, implementation, and evaluation of interventions aimed at promoting healthier behaviors across several contexts.

## Supporting information

S1 Fig Visual nudge stimulus setup Setup of the vending machine replica with the adjacent visual sensory nudge stimulus (nature-themed image). For copyright reasons, the products have been blurred.
(TIFF)

S2 Fig Olfactory nudge stimulus Materials used in the experimental conditions O: jasmine essential oil (**a**) and aroma diffuser (**b**). For copyright reasons, brands and products’ information have been blurred.
(TIFF)

S1 Table Perceived Health: internal reliability Internal reliability analysis for the perceived Health dimension of the preliminary snack classification questionnaire.
(XSXL)

S2 Table Snacks’ Perceived Health scores Perceived health scores (M) of the 26 snacks presented in the vending machine. Snacks categorized as “healthy” (above the median) are highlighted in gray. Products (11, 12, etc.) are named according to the original nomenclature of the real vending machine that was replicated. “Product Type” column specifies the category of each snack.
(XSXL)

S1 Appendix Model Fit Comparisons, Selection Criteria, and Collinearity analyses For neurophysiological data—AWI, BATR, and SC measures (Section 1) as well as for attentional data—TS and TTFF measures (Section 2).
(MS Word)
