# Supplementary figures and images for: How to nudge students toward healthier snacks? Consumer neuroscience insights on multisensory nudge interventions in university vending machines
Source: PLoS One. 2025 Jun 26;20(6):e0325804. doi: 10.1371/journal.pone.0325804 (PMC12200691; doi:10.1371/journal.pone.0325804)

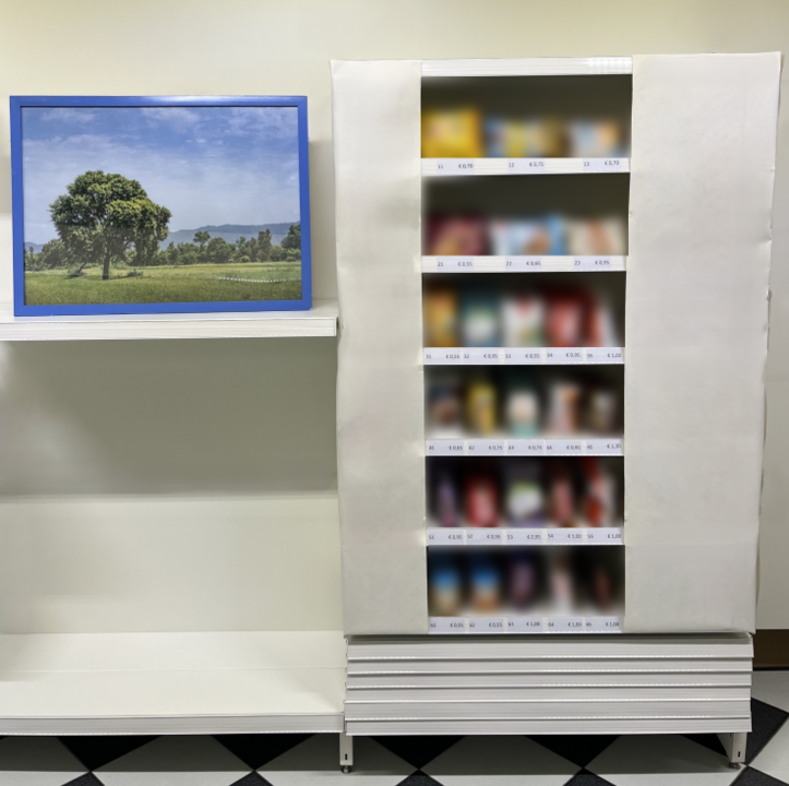

Supplement: S1 Fig — Setup of the vending machine replica with the adjacent visual sensory nudge stimulus (nature-themed image). For copyright reasons, the products have been blurred. (TIFF) [file pone.0325804.s001.tif]

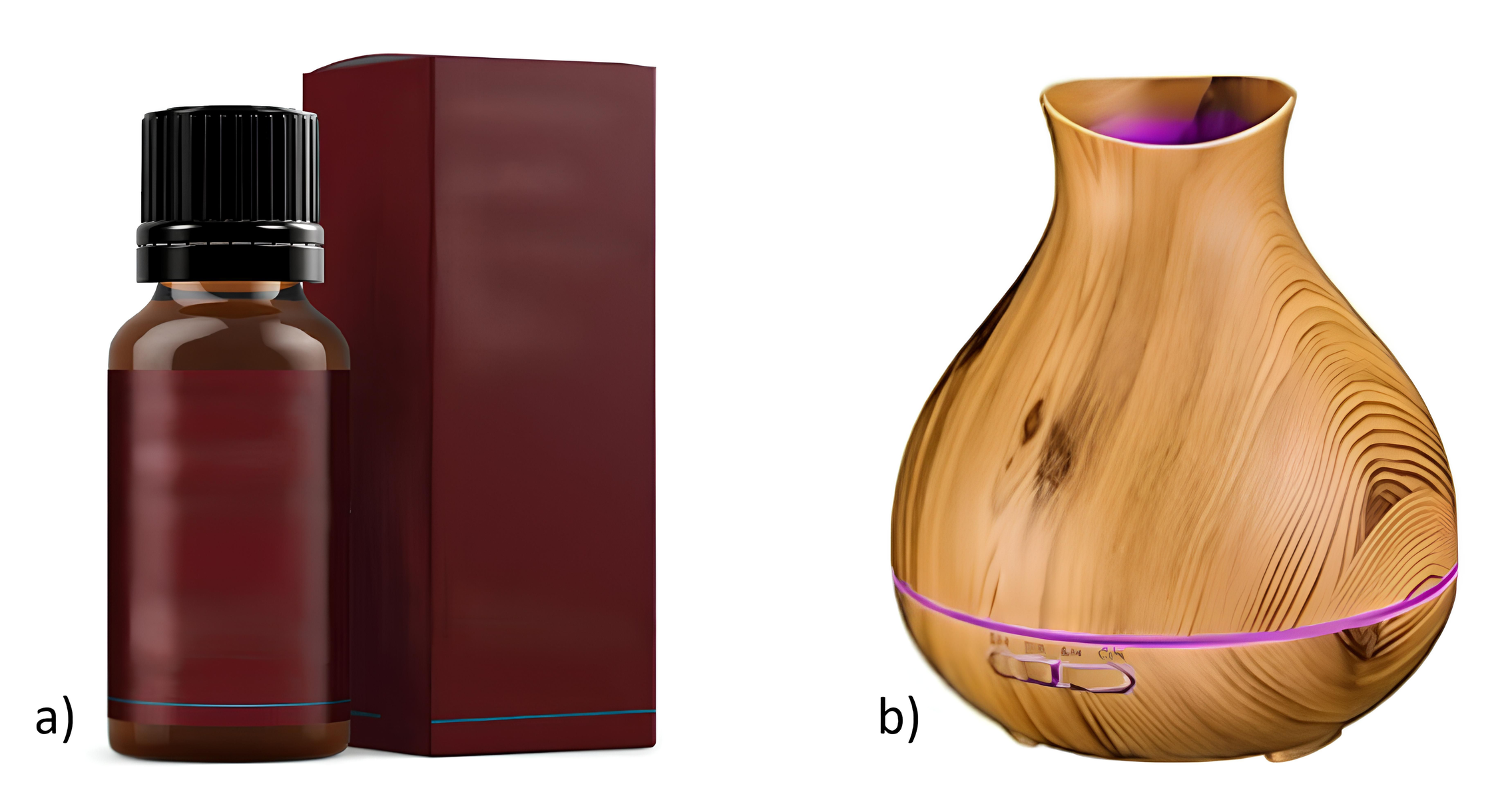

Supplement: S2 Fig — Materials used in the experimental conditions O: jasmine essential oil (a) and aroma diffuser (b). For copyright reasons, brands and products’ information have been blurred. (TIFF) [file pone.0325804.s002.tif]
