# Supplementary material for: How to nudge students toward healthier snacks? Consumer neuroscience insights on multisensory nudge interventions in university vending machines
Source: PLoS One. 2025 Jun 26;20(6):e0325804. doi: 10.1371/journal.pone.0325804 (PMC12200691; doi:10.1371/journal.pone.0325804)
Supplement: S1 Appendix — For neurophysiological data—AWI, BATR, and SC measures (Section 1) as well as for attentional data—TS and TTFF measures (Section 2). (MS Word) [file pone.0325804.s005.docx]

**Appendix**

**Section 1. Model Fit Comparison, Selection Criteria, and Collinearity of Neurophysiological Measures**

This section outlines the procedure to select the optimal model for each dependent variable y (AWI, BATR, SC)

Four models were tested for each y variable according to their respective theoretical frameworks.

- y_m1: y = 1 + (1|sbj) *[Null model]*
- y_m2: y = Condition + (1|sbj)
- y_m3: y = Condition + Phase + (1|sbj)
- y_m4: y = Condition*Phase + (1|sbj) *[Full model]*

**Approach Withdrawal Index (AWI)**

| *Model* | *npar* | *AIC* | *BIC* | *Chisq* | *Df* | *Pr*  *(>Chisq)* | *SD Intercept* | *SD Residuals* |
| --- | --- | --- | --- | --- | --- | --- | --- | --- |
| *AWI_m1* | 3 | 301.23 | 310.42 |  |  |  |  |  |
| *AWI_m2* | 6 | 301.48 | 319.86 | 5.7497 | 3 | 0.124441 | 0.389 | 0.503 |
| *AWI_m3* | 7 | 300.06 | 321.50 | 3.4221 | 1 | 0.064330 | 0.395 | 0.494 |
| *AWI_m4* | 10 | 293.33 | 323.96 | 12.7278 | 3 | 0.005264*** | 0.413 | 0.464 |

**Beta on Alpha and Theta Ratio (BATR)**

| *Model* | *npar* | *AIC* | *BIC* | *Chisq* | *Df* | *Pr*  *(>Chisq)* | *SD Intercept* | *SD Residuals* |
| --- | --- | --- | --- | --- | --- | --- | --- | --- |
| *BATR_m1* | 3 | 616.87 | 626.11 |  |  |  |  |  |
| *BATR_m2* | 6 | 622.55 | 641.04 | 0.3192 | 3 | 0.9564 | 1.613 | 1.041 |
| *BATR_m3* | 7 | 608.22 | 629.79 | 16.3320 | 1 | <0.001*** | 1.674 | 0.938 |
| *BATR_m4* | 10 | 611.17 | 641.99 | 3.0431 | 3 | 0.3850 | 1.678 | 0.937 |

**Skin Conductance (SC)**

| *Model* | *npar* | *AIC* | *BIC* | *Chisq* | *Df* | *Pr*  *(>Chisq)* | *SD Intercept* | *SD Residuals* |
| --- | --- | --- | --- | --- | --- | --- | --- | --- |
| *SC_m1* | 3 | 817.19 | 826.07 |  |  |  |  |  |
| *SC_m2* | 6 | 816.96 | 834.74 | 6.2216 | 3 | 0.10131 | 7.444 | 1.508 |
| *SC_m3* | 7 | 813.48 | 834.22 | 5.4859 | 1 | 0.01917* | 7.473 | 1.455 |
| *SC_m4* | 10 | 816.86 | 846.48 | 2.6624 | 3 | 0.45358 | 7.454 | 1.465 |

**Section 2. Model Fit Comparison, Selection Criteria, and Collinearity of Neurophysiological Measures**

This section outlines the procedure to select the optimal model for each dependent variable y (TS and TTFF)

Five models were tested for each y variable according to their respective theoretical frameworks.

- y_m1: y = 1 + (1|sbj) [*Null model*]
- y_m2: y = Shelf + (1|sbj)
- y_m3: y = Condition + (1|sbj)
- y_m4: y = Condition + Shelf + (1|sbj)
- y_m5: y = Condition*Shelf + (1|sbj) [*Full model*]

**Time Spent (TS)**

| *Model* | *npar* | *AIC* | *BIC* | *Chisq* | *Df* | *Pr*  *(>Chisq)* | *SD Intercept* | *SD Residuals* |
| --- | --- | --- | --- | --- | --- | --- | --- | --- |
| *TS_m1* | 3 | 726.34 | 740.12 |  |  |  |  |  |
| *TS_m2* | 4 | 709.72 | 728.09 | 18.625 | 1 | <.001*** | 0.054 | 0.388 |
| *TS_m3* | 6 | 724.89 | 752.44 | 0.000 | 2 | 0.000 | 0.040 | 0.393 |
| *TS_m4* | 7 | 707.62 | 739.78 | 19.262 | 1 | <.001*** | 0.042 | 0.388 |
| *TS_m5* | 10 | 702.80 | 748.73 | 10.822 | 3 | 0.01273* | 0.027 | 0.387 |

**Time to First Fixation (TTFF)**

| *Model* | *npar* | *AIC* | *BIC* | *Chisq* | *Df* | *Pr*  *(>Chisq)* | *SD Intercept* | *SD Residuals* |
| --- | --- | --- | --- | --- | --- | --- | --- | --- |
| *TT_m1* | 3 | 3816.5 | 3830.3 |  |  |  |  |  |
| *TT_m2* | 4 | 3813.5 | 3831.9 | 4.9602 | 1 | 0.02495* | 1.259 | 1.122 |
| *TT_m3* | 6 | 3793.9 | 3821.5 | 23.6085 | 2 | <.001*** | 0.804 | 3.153 |
| *TT_m4* | 7 | 3791.5 | 3823.7 | 4.3769 | 1 | 0.03643* | 0.819 | 3.144 |
| *TT_m5* | 10 | 3788.0 | 3834.0 | 9.4911 | 3 | 0.02343* | 0.7977 | 3.132 |
